# Improving confidence in MRI-based auto-segmentation via uncertainty assessment

**DOI:** 10.2340/1651-226X.2026.45685

**Published:** 2026-05-11

**Authors:** Jesper Folsted Kallehauge, Jintao Ren, Yasmin Lassen-Ramshad

**Affiliations:** a Danish Centre for Particle Therapy, Aarhus University Hospital, Aarhus, Denmark; b Department of Clinical Medicine, Aarhus University, Aarhus, Denmark

**Keywords:** Deep learning, image segmentation, magnetic resonance imaging, uncertainty, calibration, radiotherapy planning, computer-assisted, brain neoplasms

## Abstract

**Background and purpose:**

Accurate delineation of organs of interest (OOIs, also commonly referred to as organs at risk, OARs) is crucial for safe radiotherapy. While deep learning-based segmentation using convolutional neural networks has achieved high geometric accuracy, clinical translation is hindered by overconfident, uncalibrated predictions in anatomically ambiguous regions. Uncertainty quantification and model calibration are prerequisites for safe clinical workflows. This study compared the standard nnU-Netv2 against its residual-encoding variant (ResEncM), hypothesizing that ResEncM would demonstrate superior reliability and calibration while maintaining comparable geometric accuracy.

**Patient/material and methods:**

T1-weighted contrast-enhanced MRI scans from 70 brain cancer patients were used (55 training/validation, 15 testing). Ground-truth contours for brainstem, hippocampi, chiasm, optic nerves, optic tracts, and pituitary were delineated per Danish Neuro Oncology Group guidelines. Both architectures were trained using five-fold cross-validation with identical preprocessing. Epistemic uncertainty was quantified using mutual information, and Expected Calibration Error (ECE) was computed within a 10-mm isotropic margin around reference contours.

**Results:**

Both models achieved high geometric accuracy (brainstem dice similarity coefficient [DSC] > 0.93, hippocampi DSC > 0.81). No significant geometric differences were found for large structures. ResEncM showed significantly lower DSC for the pituitary (*p* = 0.003) and chiasm (*p* = 0.018). However, ResEncM demonstrated significantly lower epistemic uncertainty and ensemble variance across all structures (*p* < 0.05), and significantly reduced ECE for the optic chiasm, optic tracts, and pituitary.

**Interpretation:**

Integrating a deep residual encoder into the standard U-Net framework significantly improves reliability and calibration of automated brain OOI contours while maintaining strong geometric performance. The ResEncM architecture provides a more trustworthy tool for clinical radiotherapy by reliably flagging high-uncertainty voxels, supporting confidence-aware clinical workflows.

## Graphical abstract

**Figure:**
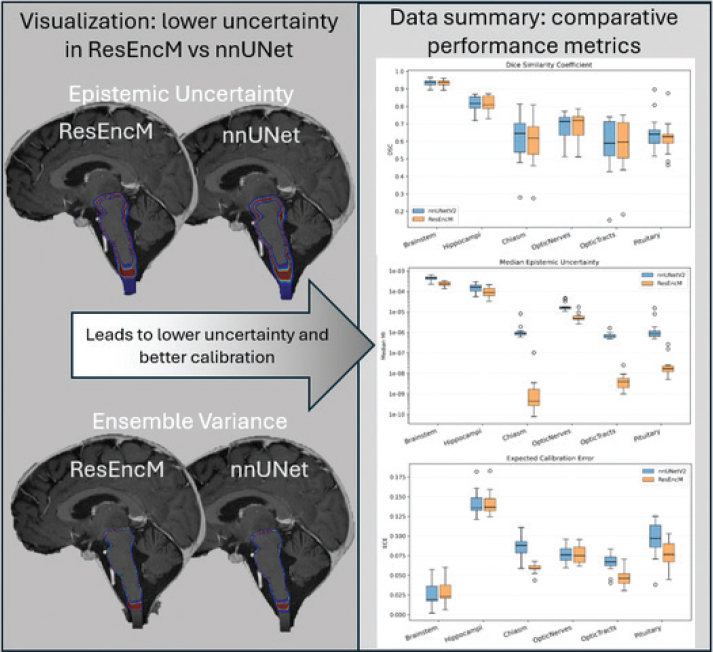

## Introduction

Accurate delineation of organs of interest (OOIs) is crucial for safe and effective radiotherapy. In the era of artificial intelligence (AI), the field has transitioned from manual contouring – a process influenced by significant inter-observer variability [1] and inefficiency – to automated segmentation using Deep Neural Networks (DNNs) [2]. Among these, the nnU-Net framework established itself as the de facto standard, owing to its self-configuring approach that robustly handles diverse medical imaging datasets [3]. However, as the clinical integration of these models accelerates, the focus of the research community has moved from pure geometric accuracy (e.g., measured by Dice Similarity Coefficients) to model reliability, calibration, and uncertainty quantification (UQ) [4].

Despite the high geometric accuracy of standard convolutional neural networks (CNNs)-based UNets in medical image segmentation, their clinical translation is frequently hindered by a tendency to produce highly overconfident and uncalibrated predictions in anatomically ambiguous regions. To address this safety concern, recent literature heavily emphasizes UQ as a prerequisite for safe human-in-the-loop AI radiotherapy workflows [5]. UQ fundamentally distinguishes between two sources of doubt: aleatoric uncertainty, which arises from inherent data noise such as MRI artifacts or ambiguous tissue contrast, and epistemic uncertainty, which represents model ignorance and can be mitigated in part by architectural improvements or additional training data [6]. To capture this epistemic doubt, Deep Ensembles – which aggregate predictions across multiple independently trained models – have become a widely adopted strategy. They frequently demonstrate more reliable uncertainty estimation than single deterministic networks and serve as a highly robust alternative to Monte Carlo Dropout approximations [7]. Furthermore, the field is increasingly prioritizing model calibration, measured via Expected Calibration Error (ECE) [7], to ensure that a network’s output softmax probabilities accurately reflect its true likelihood of correctness. In simpler terms, calibration ensures that if the AI is 90% confident in a treatment boundary, that boundary is actually correct 90% of the time, preventing dangerous overconfidence. However, while standard U-Net architectures typically achieve high spatial overlap metrics, they often lack the internal mechanisms to maintain calibration at complex boundaries, motivating the exploration of deeper, feature-rich architectures to inherently improve model reliability [8].

Recent architectural advancements have introduced residual-encoding variants (such as ResEncM) to the standard nnU-Net framework. While residual connections are known to improve feature extraction and gradient flow, their specific impact on model certainty, predictive stability, and probability calibration in small or complex brain structures remains under-investigated.

Therefore, this study compared the standard nnUNet (v2) against its residual-encoding variant (ResEncM) [9]. We hypothesized that while ResEncM would maintain comparable geometric accuracy to the standard nnU-Netv2, it would demonstrate superior reliability and calibration.

## Patients/material and methods

### Patient data and image acquisition

The study included T1-weighted contrast-enhanced MRI scans from 70 adult patients with brain cancer, acquired on a 3T MRI scanner (Philips Ingenia) at a single institution. The dataset was partitioned into 55 patients for training/validation and 15 patients for independent testing. Ground-truth contours for OOIs – including the brainstem, hippocampi, chiasm, optic nerves, optic tracts, and pituitary – were delineated according to Danish Neuro Oncology Group (DNOG) consensus guidelines [10].

#### Model architecture and training

We configured a baseline 3D U-Net (nnU-Netv2) architecture and a residual-encoding variant (ResEncM) for this study. For both models, the input included a single modality channel (T1-weighted contrast-enhanced MRI) with an input patch size set to 128 × 128 × 128. The baseline nnU-Netv2 consisted of six depth levels (32, 64, 128, 256, 320, and 320 feature channels, respectively), utilizing two standard convolutional operations per encoder and decoder stage.

To evaluate the impact of architectural modifications, the ResEncM model variant maintained the identical input patch size, depth levels, and feature channel distribution to ensure a controlled comparison. However, its standard convolutional encoder was replaced with a Residual Encoder. Specifically, the ResEncM encoder stages utilized an increasing number of residual blocks (1, 3, 4, 6, 6, and 6 blocks across the respective depth levels) to enhance feature propagation, while the decoder was streamlined to utilize a single convolutional operation per stage.

Both architectures were trained using identical preprocessing pipelines, data augmentations, and a five-fold cross-validation strategy. All five folds contributed to the final ensemble predictions for each test patient.

### Uncertainty and calibration assessment

Epistemic uncertainty was quantified voxel-wise using mutual information, and ensemble variance was derived from the variance of the softmax probabilities across the model folds. To evaluate calibration, the ECE was computed per structure using fold-averaged softmax outputs. ECE calculations were restricted to a 10-mm isotropic margin around the reference contours to focus the metric on the clinically relevant organ boundaries.

### Geometric accuracy and statistical analysis

Geometric accuracy was evaluated using the Dice similarity coefficient (DSC) and 1-mm Normalized Surface Dice (1NSD). Differences in accuracy, uncertainty, and calibration metrics between nnU-Netv2 and ResEncM were assessed using paired Wilcoxon signed-rank tests, with statistical significance defined as *p* < 0.05.

## Results

### Geometric accuracy

Both the baseline nnU-Netv2 and the ResEncM architectures achieved high geometric accuracy across the evaluated OOIs. Median DSC exceeded 0.93 for the brainstem and 0.81 for the hippocampi for both models (Table 1). Statistical analysis revealed no significant differences in geometric accuracy (DSC and 1-mm Normalized Surface Dice) between the two architectures for the majority of the large-volume structures. However, a slight performance shift was observed for small, highly variable anatomies; the ResEncM model demonstrated significantly lower DSC and 1NSD compared to the baseline nnU-Netv2 for both the pituitary gland (*p* = 0.003) and the optic chiasm (DSC *p* = 0.018, 1NSD *p* = 0.005) (Table 1).

**Table 1:** Comparison of geometric accuracy (DSC, 1NSD) and uncertainty/calibration metrics (Epistemic MI, Ensemble Variance, ECE) between the baseline nnU-Netv2 and the ResEncM architecture across brain organs of interest.

Metric / model	Brainstem	Hippocampi	Optic chiasm	Optic nerves	Optic tracts	Pituitary
**DSC median (Range)**						
**nnU-Netv2**	0.94 (0.89–0.97)	0.82 (0.72–0.87)	0.63 (0.28–0.81)	0.71 (0.42–0.77)	0.56 (0.15–0.74)	0.64 (0.51–0.90)
**ResEncM**	0.94 (0.89–0.96)	0.81 (0.73–0.87)	0.61 (0.28–0.81)	0.72 (0.45–0.79)	0.58 (0.18–0.75)	0.62 (0.47–0.88)
***p-value***	*0.561*	*0.561*	***0.018***	*0.389*	*0.679*	***0.003***
**1NSD median (range)**						
**nnU-Netv2**	0.88 (0.71–0.97)	0.90 (0.74–0.95)	0.77 (0.53–0.98)	0.85 (0.56–0.97)	0.86 (0.31–0.98)	0.68 (0.37–1.00)
**ResEncM**	0.88 (0.72–0.97)	0.92 (0.75–0.96)	0.77 (0.50–0.97)	0.86 (0.57–0.98)	0.82 (0.36–0.95)	0.63 (0.35–1.00)
***p-value***	*0.890*	*0.454*	***0.005***	*0.561*	*0.804*	***0.003***
**Epistemic MI Median (Range)**						
**nnU-Netv2**	4.6e-4 (2.2e-4–6.5e-4)	1.6e-4 (5.5e-5–3.0e-4)	8.8e-7 (6.0e-7–8.1e-6)	1.6e-5 (1.1e-5–4.7e-5)	6.5e-7 (4.8e-7–1.6e-6)	8.7e-7 (4.8e-7–1.5e-5)
**ResEncM**	2.4e-4 (1.4e-4–3.2e-4)	8.9e-5 (3.3e-5–2.1e-4)	4.4e-10 (7.7e-11–1.0e-7)	4.7e-6 (2.6e-6–1.7e-5)	3.9e-9 (9.7e-10–2.5e-8)	1.7e-8 (5.0e-9–2.6e-7)
***p-value***	***< 0.001***	***< 0.001***	***< 0.001***	***< 0.001***	***< 0.001***	***< 0.001***
**Ens. Variance Median (Range)**						
**nnU-Netv2**	3.2e-9 (5.8e-10–8.0e-9)	2.6e-11 (1.9e-12–1.6e-10)	4.1e-16 (1.6e-16–3.6e-14)	2.0e-13 (8.9e-14–2.1e-12)	1.6e-16 (7.6e-17–1.1e-15)	4.2e-16 (7.8e-17–3.1e-13)
**ResEncM**	9.8e-10 (2.7e-10–2.6e-9)	1.4e-11 (9.6e-13–7.3e-11)	2.8e-22 (8.6e-24–1.1e-17)	2.5e-14 (8.1e-15–4.4e-13)	1.7e-20 (1.4e-21–7.0e-19)	2.5e-19 (2.3e-20–7.2e-17)
***p-value***	***< 0.001***	***0.049***	***< 0.001***	***< 0.001***	***< 0.001***	***< 0.001***
**ECE Median**						
**nnU-Netv2**	0.02 (0.00–0.06)	0.14(0.12–0.18)	0.09(0.06–0.11)	0.08 (0.06–0.10)	0.07 (0.04–0.08)	0.10 (0.04–0.13)
**ResEncM**	0.02 (0.01–0.06)	0.14 (0.12–0.18)	0.06 (0.04–0.07)	0.08 (0.06–0.10)	0.05 (0.03–0.07)	0.08 (0.04–0.10)
***p-value***	***0.001***	*0.391*	***< 0.001***	*0.358*	***< 0.001***	***0.005***

DSC: dice similarity coefficient; 1NSD: 1-mm normalized surface dice; MI: mutual information; Ens. Variance: ensemble variance; ECE: expected calibration error.

Bold, italicized values indicate statistical significance (*p* < 0.05) based on paired Wilcoxon signed-rank tests (*n* = 14 pairs for uncertainty and ECE metrics, *n* = 15 for geometric metrics).

### Uncertainty and calibration

**Figure 1:**
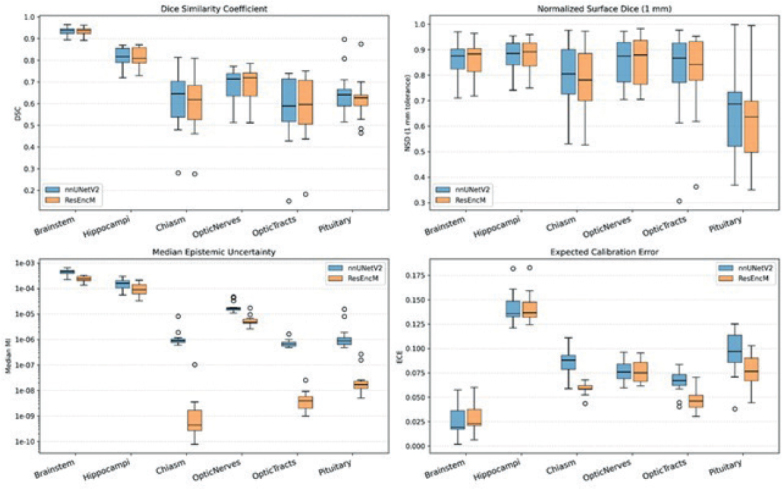
Quantitative impact of residual encoding on epistemic uncertainty and calibration for brain organs of interest. Box-and-whisker plots comparing prediction accuracy: Dice Similarity Coefficient (upper left) and 1 mm normalized surface Dice (upper right) and uncertainty: Epistemic Mutual Information (lower left), and Expected Calibration Error (ECE) lower right between the baseline nnU-Netv2 (blue) and ResEncM (orange) architectures for the optic chiasm, optic tracts, and pituitary gland.

**Figure 2:**
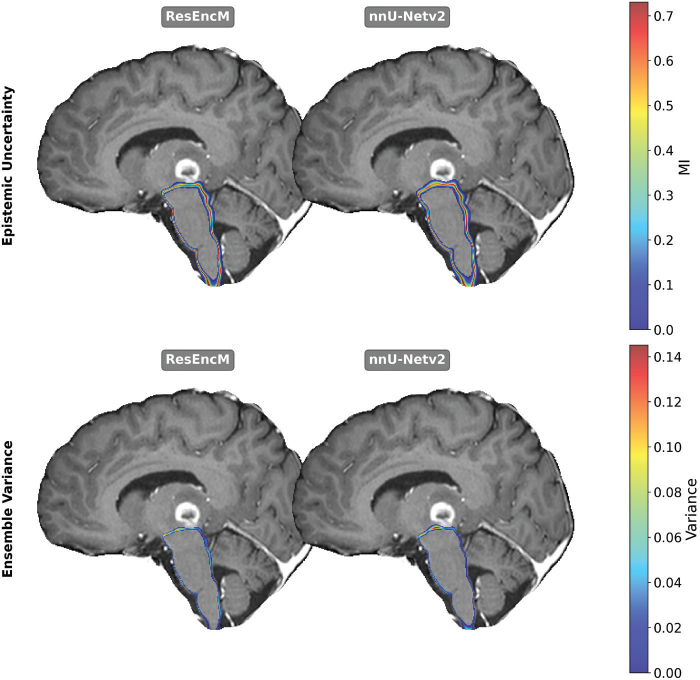
Comparison of ResEncM and nnUNet epistemic uncertainty for brainstem (top) and Ensemble Variance (bottom). Slightly tighter values around the brainstem were seen for ResEncM compared to nnUNet for both metrics.

Despite the localized reductions in spatial overlap for small structures, the integration of residual encoding yielded marked improvements in model reliability and confidence. The ResEncM architecture demonstrated consistently and significantly lower epistemic uncertainty and ensemble variance across all evaluated structures (all *p* < 0.05; see Table 1 for individual *p*-values) (Table 1). These reductions were most pronounced for small, low-contrast anatomies, with the largest quantitative improvements observed in the optic chiasm, optic tracts, and pituitary gland (Figure 1). Qualitative visual analysis confirmed this finding, revealing a tighter, less diffuse uncertainty profile strictly along the OOI boundaries for the ResEncM model compared to the standard nnU-Netv2 (Figure 2). A comprehensive comparison of epistemic uncertainty and ensemble variance maps for all six OOIs is provided in Supplementary Figures 1 and 2, respectively.

Furthermore, the ECE, computed within a 10-mm isotropic margin around the reference contours, was significantly reduced by the ResEncM architecture for the optic chiasm, optic tracts, and pituitary gland (Table 1). This indicates a much better alignment between the predicted softmax probabilities and true segmentation correctness in these challenging regions. ECE values did not show significant differences between the two models for the hippocampi and optic nerves. Conversely, the brainstem exhibited a slight but statistically significant increase in ECE with the ResEncM model (*p* = 0.001), though absolute calibration remained exceptionally high (median ECE 0.02) (Table 1). Overall, these metrics indicated that the ResEncM model provided superior calibration and drastically lower epistemic doubt for challenging structures, effectively substituting the diffuse, uncertain predictions of the baseline model with tightly calibrated contours.

## Discussion and conclusion

The clinical translation of deep learning for radiotherapy segmentation requires models that are not only geometrically accurate but also robustly calibrated. In this study, we demonstrated that modifying the standard nnU-Netv2 architecture with a residual encoder (ResEncM) significantly reduced epistemic uncertainty and ECE in brain MRI segmentation without broadly compromising geometric accuracy.

Importantly, the baseline geometric accuracy achieved in this study is highly consistent with current state-of-the-art clinical benchmarks. For large, high-contrast structures like the brainstem and hippocampi, our models’ median DSCs (> 0.93 and > 0.81, respectively) align with or exceed the performance of established commercial models and previous nnU-Net implementations, which typically report brainstem DSCs of 0.86–0.89 and hippocampal DSCs of 0.78–0.80. Similarly, for small, difficult structures like the optic chiasm (DSC ~0.61–0.63) and optic nerves (DSC ~0.71), our spatial overlap is on par with recent literature benchmarks that report DSCs ranging from 0.49 to 0.63 for the chiasm and 0.66 to 0.70 for the optic nerves [11].

Building upon this strong geometric foundation, our findings align with recent literature emphasizing the critical shift from pure geometric evaluation toward UQ to ensure clinical safety. Ren et al. [12] highlighted that standard convolutional networks often produce overconfident, uncalibrated predictions in challenging head and neck boundary regions. Similarly, our baseline nnU-Netv2 exhibited diffuse and overconfident epistemic doubt at the boundaries of small, low-contrast structures like the optic chiasm and optic tracts.

By replacing the standard encoder with an asymmetric, heavy encoder, light decoder residual architecture, the ResEncM model effectively mitigated this epistemic doubt. As established by Ghaffari et al. [13] and Kumar et al. [14], utilizing an increasing number of residual blocks at the deepest spatial levels maximizes hierarchical feature extraction and prevents vanishing gradients. This architectural depth allowed the ResEncM network to build a vastly more robust internal representation of complex anatomical relationships before spatial upsampling, granting it the mathematical capacity to appropriately calibrate its confidence in ambiguous regions.

Interestingly, while ResEncM improved calibration across most structures, it yielded a significantly lower DSC for the pituitary gland and optic chiasm. This highlights a critical trade-off between geometric hedging and mathematical calibration. Standard U-Net architectures frequently achieve high spatial overlap by generating diffuse boundaries in ambiguous regions – inadvertently capturing more of the subjective ground truth while outputting untrustworthy probabilities. In contrast, the deep residual encoder forces the network to make sharp, highly calibrated decisions. While this strict decisiveness penalizes Dice scores in small, highly variable structures, it drastically reduces epistemic uncertainty. Conversely, for structurally obvious organs like the brainstem, the baseline nnU-Netv2 was already exceptionally well-calibrated. The slight increase in ECE observed with the ResEncM model for the brainstem suggests that while deep residual encoding is vital for resolving highly ambiguous anatomies, it may lead to slight over-parameterization for simpler segmentation tasks.

A major strength of this study is the highly controlled experimental design; by utilizing the nnU-Netv2 self-configuring framework, we ensured that all preprocessing, patch sizes, and augmentations were completely identical, isolating the residual encoder as the sole driver of the improved ECE and uncertainty metrics. Furthermore, evaluating calibration within a 10-mm isotropic margin focused our analytical metrics strictly on the clinically relevant organ boundaries rather than the easily classified background.

However, several limitations regarding data quality and scope must be acknowledged. The dataset was restricted to 70 patients from a single institution, utilizing solely T1-weighted contrast-enhanced MRI scans. This single-center approach limits generalizability, as multi-institutional data with varying MRI acquisition protocols (or multi-parametric MRI) might influence baseline aleatoric noise and subsequent calibration. Additionally, the relatively small test cohort (*n* = 15) limits the statistical power of the anatomical subgroup analyses. Finally, our epistemic uncertainty estimation relies on a standard 5-fold cross-validation ensemble. While practical for standard training pipelines, an ensemble of only five models may be insufficient to fully and accurately capture the true underlying prediction variance, potentially limiting the precision of our uncertainty estimates. Notably, the current model is limited to the intracranial OOIs defined by Danish Neuro Oncology Group (DNOG) guidelines that are delineated on T1-weighted contrast-enhanced MRI; future work should incorporate additional imaging modalities to enable segmentation of the full guideline-defined structure set.

Furthermore, while this study focused on geometric and probabilistic evaluation metrics, we acknowledge that the ultimate validation of auto-segmentation tools lies in their dosimetric and clinical impact. Future studies should evaluate how uncertainty-guided workflows affect treatment plan quality, clinical acceptability of auto-contours, physician editing time, and whether uncertainty maps effectively direct clinician review to regions requiring manual correction.

While this study primarily evaluated output metrics, the disparity in calibration between the two architectures likely relates to their fundamental handling of spatial data. One limitation of the study is a missing internal feature analysis. Standard nnU-Netv2 employs sequential convolutional blocks that progressively compress information, whereas the ResEncM encoder variant utilizes residual ‘identity mappings’ to preserve high-resolution features from earlier layers. The superior ECE of ResEncM therefore suggests that this structural preservation of fine-grained detail may enable more reliable probability estimation at complex anatomical transition zones, where the standard architecture may be more prone to over-smoothing or information loss.

In conclusion, the integration of a deep residual encoder into the standard U-Net framework significantly improves the reliability and calibration of automated OOI contours in brain MRI. By reducing epistemic uncertainty and ECE while maintaining strong geometric performance, the ResEncM architecture provides a more transparent and trustworthy tool for clinical radiotherapy. By reliably flagging high-uncertainty voxels, this approach supports a safe, ‘confidence-aware’ workflow, effectively directing the clinician’s manual review to areas where the AI’s internal doubt is justified.

## Supplementary Material



## Data Availability

The clinical datasets analyzed during the current study are not publicly available due to patient privacy and institutional data protection policies. However, the trained segmentation models and the evaluation scripts used in this study are available from the corresponding author upon reasonable request.
