# Supplementary material for: Improving confidence in MRI-based auto-segmentation via uncertainty assessment
Source: Acta Oncol. 2026 May 11;65:45685. doi: 10.2340/1651-226X.2026.45685 (PMC13173403; doi:10.2340/1651-226X.2026.45685)

Supplementary material has been published as submitted. It has not been copyedited, or typeset by Acta Oncologica

Supplementary Figure 1: Epistemic Uncertainty (Mutual Information)
nnU-Netv2 vs ResEncM – All Structures

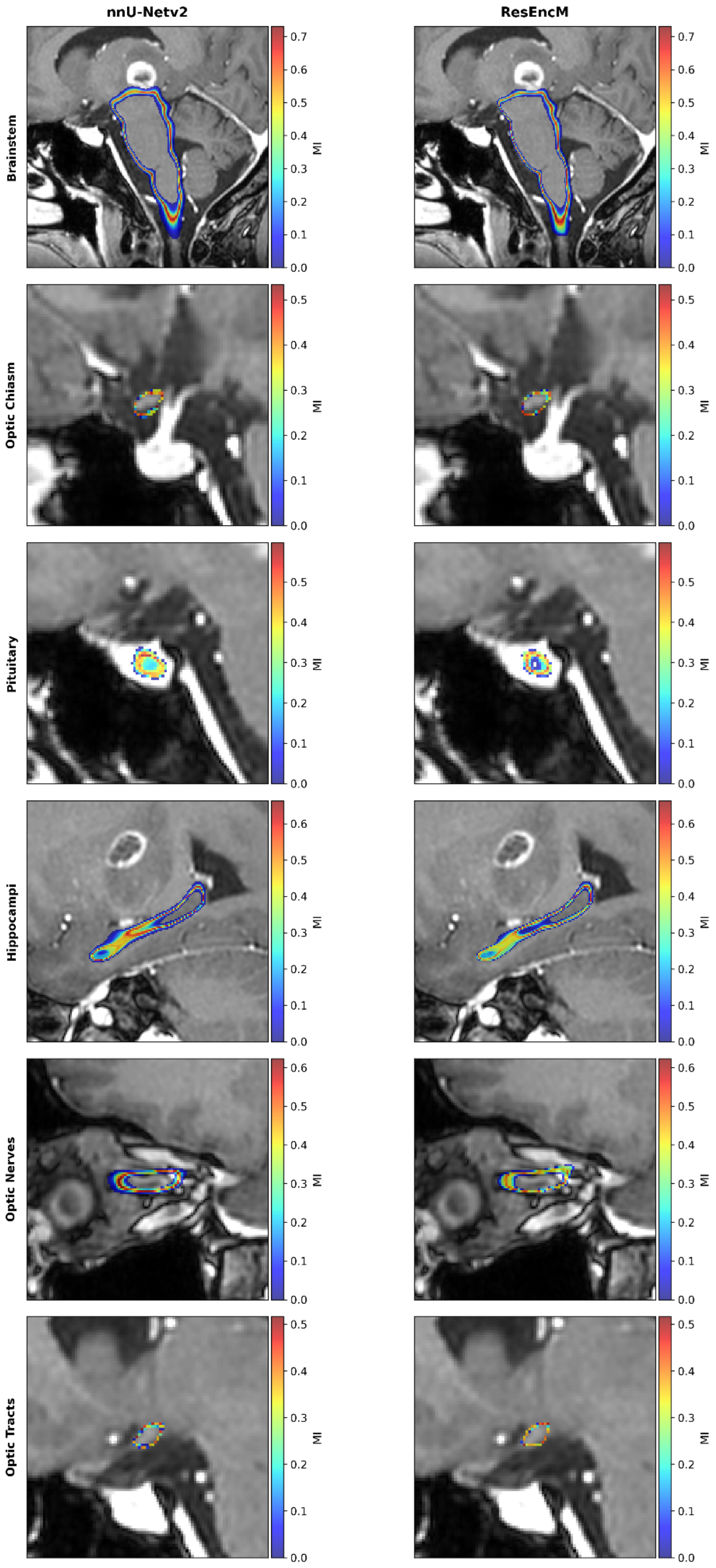

Supplementary Figure 2: Ensemble Variance
nnU-Netv2 vs ResEncM – All Structures

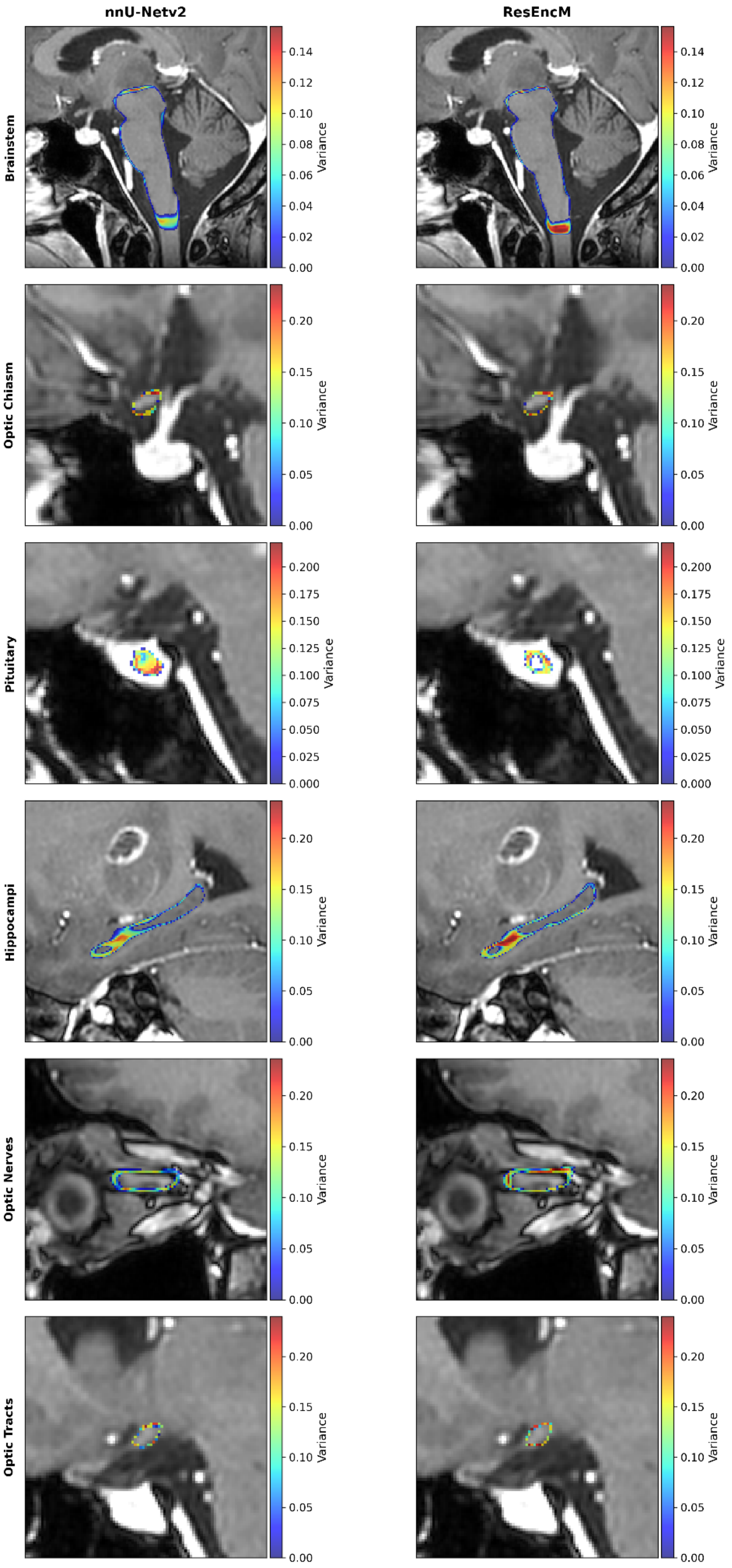

Supplement: Supplementary file 1 [file AO-65-45685-s1.pdf]
